# Effect of Immunosuppressive Agents on Hepatocyte Apoptosis Post-Liver Transplantation

**DOI:** 10.1371/journal.pone.0138522

**Published:** 2015-09-21

**Authors:** Eu Jin Lim, Ruth Chin, Ueli Nachbur, John Silke, Zhiyuan Jia, Peter W. Angus, Joseph Torresi

**Affiliations:** 1 Liver transplant unit, Austin Hospital, Heidelberg, VIC, Australia; 2 Department of Medicine, The University of Melbourne, Austin Hospital, Heidelberg, VIC, Australia; 3 Walter and Eliza Hall Institute, Parkville, VIC, Australia; 4 Department of Infectious Diseases, Austin Hospital, Heidelberg, VIC, Australia; 5 Department of Microbiology and Immunology, The Peter Doherty Institute for Infection and Immunity, The University of Melbourne, Parkville, VIC, Australia; Martin Luther University, GERMANY

## Abstract

**Introduction:**

Immunosuppressants are used ubiquitously post-liver transplantation to prevent allograft rejection. However their effects on hepatocytes are unknown. Experimental data from non-liver cells indicate that immunosuppressants may promote cell death thereby driving an inflammatory response that promotes fibrosis and raises concerns that a similar effect may occur within the liver. We evaluated apoptosis within the liver tissue of post-liver transplant patients and correlated these findings with *in vitro* experiments investigating the effects of immunosuppressants on apoptosis in primary hepatocytes.

**Methods:**

Hepatocyte apoptosis was assessed using immunohistochemistry for M30 CytoDEATH and cleaved PARP in human liver tissue. Primary mouse hepatocytes were treated with various combinations of cyclosporine, tacrolimus, sirolimus, or MMF. Cell viability and apoptosis were evaluated using crystal violet assays and Western immunoblots probed for cleaved PARP and cleaved caspase 3.

**Results:**

Post-liver transplant patients had a 4.9-fold and 1.7-fold increase in M30 CytoDEATH and cleaved PARP compared to normal subjects. Cyclosporine and tacrolimus at therapeutic concentrations did not affect hepatocyte apoptosis, however when they were combined with MMF, cell death was significantly enhanced. Cell viability was reduced by 46% and 41%, cleaved PARP was increased 2.6-fold and 2.2-fold, and cleaved caspase 3 increased 2.2-fold and 1.8-fold following treatment with Cyclosporine/MMF and Tacrolimus/MMF respectively. By contrast, the sirolimus/MMF combination did not significantly reduce hepatocyte viability or promote apoptosis.

**Conclusion:**

Commonly used immunosuppressive drug regimens employed after liver transplantation enhance hepatocyte cell death and may thus contribute to the increased liver fibrosis that occurs in a proportion of liver transplant recipients.

## Introduction

Immunosuppressive agents are used after liver transplantation in order to prevent rejection of the transplanted allograft. The mechanisms by which these immunosuppressive agents exert their effects are varied. Cyclosporine and tacrolimus are potent immunosuppressive agents that bind to cyclophillin, resulting in the inhibition of calcineurin, a key enzyme required for IL-2 production in T-cells, thereby blocking the recruitment and activation of CD4 T-cells[1]. In clinical trials, tacrolimus has been found to be superior to cyclosporine in preventing acute rejection, graft loss, and postoperative death[2]. In contrast, sirolimus is an mTOR inhibitor, which exerts its immunosuppressive effect by blocking the proliferation and clonal expansion of antigen-activated T-cells[3]. Mycophenolic acid, the active metabolite of mycophenolate mofetil (MMF), has a different mechanism of action involving the inhibition of inosine monophosphate dehydrogenase, blocking de novo purine synthesis which is required for lymphocyte proliferation[4]. Immunosuppressive regimens consisting of a combination of MMF and a calcineurin inhibitor, or more recently sirolimus, are commonly used for maintenance immunosuppression following liver transplantation.

After transplantation for hepatitis C (HCV) disease, patients often have more aggressive liver disease than in the non-transplant setting, with 20% of transplant recipients with HCV recurrence progressing to cirrhosis within 5 years of liver transplantation[5]. Hepatocyte apoptosis has been found to be more pronounced in the livers of HCV-infected patients post-liver transplantation compared to patients with chronic HCV[6], indicating that the immunosuppressants used may promote liver injury.

Despite their universal use, the effect of these immunosuppressive agents on hepatocyte viability and apoptosis is unknown. In non-liver cell types these agents have been shown to enhance cell death[7–10]. But whether they have similar effects in hepatocytes and thus may contribute to the pathogenesis of allograft injury post-liver transplant is unknown.

In this study, we have evaluated hepatocyte cell death within the liver tissue of patients on immunosuppressants post liver transplant and compared this to the liver tissue of normal individuals without liver disease. In addition, we correlated these findings with *in vitro* experiments investigating the effects of cyclosporine, tacrolimus, sirolimus and MMF alone and in combination on cell death of primary hepatocytes.

## Materials and Methods

### Immunohistochemistry of human liver specimens

Human liver tissue was stained for the markers of apoptosis cleaved cytokeratin 18 (M30 CytoDEATH, Enzo Life Sciences) and cleaved PARP (Cell Signaling Technology). Immunohistochemistry was performed as previously described[11]. In brief, 4 μm sections of paraffin-embedded human liver tissue mounted on silane-coated glass slides were de-paraffinized in histolene and dehydrated in graded ethanol. Endogenous peroxidase activity was blocked with 3% hydrogen peroxide in PBS. Non-specific proteins were blocked with Protein Block Serum-free (DakoCytomation) for 30 minutes at room temperature. Blocked tissues were incubated overnight at 4°C with either M30 CytoDEATH or cleaved PARP antibody, 1:100 in diluent as directed by the manufacturer. The following day, sections were incubated with their respective biotinylated-conjugated secondary antibody (1:200) for 1 hour at room temperature, followed by incubation with avidin–biotin Vectastain ABC system (Vector Laboratories) for 30 minutes. Diaminobenzidine tetrahydrochloride (DAB, Sigma-Aldrich) was then added as a chromogen and sections counterstained in haematoxylin. The relative staining in each group was assessed by computerized image capture quantification using the MCID Analysis software (InterFocus Imaging) and the results expressed as the proportional area stained, which is the proportion of cells staining positive in the given area.

### Preparation of Primary mouse Hepatocytes

Primary mouse hepatocytes (PMoH) were isolated from up to 12-week-old C57BL/6 mice, as previously described[12]. In brief, PMoH were extracted via ex-vivo perfusion of the left liver lobe with Ca^2+^ and Mg^2+^ free HEPES buffer (Invitrogen), followed by HEPES containing 500 mg/l collagenase IV (Sigma-Aldrich). Hepatocytes were separated on a 45% Percoll density gradient (Sigma-Aldrich) and seeded at a density of 50,000 cells/cm^2^ in cell culture plates coated with 0.6 mg/ml rat-tail collagen (Sigma-Aldrich). PMoH were cultured overnight in complete Williams E medium (Invitrogen) containing 10% FBS, 1% glutamine (Sigma-Aldrich), 50μg/ml penicillin/streptomycin (Invitrogen) and 1:1000 gentamicin (Invitrogen). Prior to infection with recombinant adenoviruses, non-adherent cells were washed off and adherent cells incubated with complete Williams E medium supplemented with 1% HEPES pH 7.4 (Invitrogen), 0.1% gentamicin (Invitrogen), 1% glutamine (Invitrogen), 1% linoleic acid (Sigma-Aldrich), 1% epidermal growth factor (EGF) (BD BioScience), 0.1% ITS (Sigma-Aldrich), 0.1% insulin (Sigma-Aldrich), 0.01% dexamethasone (Sigma-Aldrich) and 0.01% ethanolamine (Sigma-Aldrich).

### Hepatocyte treatment with immunosuppressive agents

PMoH were grown for 6 hours before being treated with immunosuppressive agents. Time course experiments were performed with the therapeutically relevant concentrations of these agents used post-liver transplantation to determine their effect on hepatocyte cell death. All experiments were performed in triplicate.

Cyclosporine (Neoral^TM^; 100 mg/ml stock solution, provided as a gift from Novartis) was diluted to 1 μg/ml. Tacrolimus (FK-506, F4679; Sigma) was reconstituted to form a working solution of 0.005, μg/ml. Sirolimus (Rapamycin, R8781; 2.5 mg/ml stock solution, Sigma) was serially diluted into 0.01 μg/ml. MMF (Cellcept^TM^; provided as a gift from Roche) was reconstituted into a working solution of 5 μg/ml.

Combinations of drugs were selected to mimic immunosuppressant regimens commonly used in the post-liver transplant setting. For these experiments, the therapeutically relevant concentrations of 1 μg/ml of cyclosporine, 0.005 μg/ml of tacrolimus, 0.01 μg/ml of sirolimus, and 5 μg/ml of MMF were used.

### Western immunoblot detection of cellular proteins

PMoH were harvested at 48 hr post-treatment in 200 μl ice-cold cell lysis buffer (Cell Signaling Technology) supplemented with 1 mM sodium molybdate, 5 mM sodium fluoride, 1M DTT (Sigma-Aldrich) and 1x Complete Protease Inhibitor (Roche). Thirty micrograms of total cytoplasmic proteins were resolved by 12% denaturing SDS-PAGE gel, transferred into Hybond-C Extra membrane (GE Healthcare) and analyzed by immunoblotting. Markers of apoptosis were detected using anti-caspase 3 (Cell Signaling Technology) and anti-cleaved PARP (poly ADP-ribose polymerase, Abcam) antibodies. Anti-pan-actin was used as a loading control. Immunoblots were analyzed with the BioRad GS800 densitometer using the Quantity One software and protein bands of interest were corrected for background activation.

### Cell Viability assays

Cell viability was determined using a crystal violet assay. PMoH (2.5 x 10^5^ cells/well) were seeded in 12-well plates and treated with various concentrations of the immunosuppressants. At 24, 48 and 72 hr post-treatment, wells were washed twice with sterile PBS and cells were fixed and stained with 0.1% crystal violet in 1M citric acid containing 20% methanol for 20 minutes at room temperature. Wells were washed thoroughly with sterile PBS to remove excess crystal violet and then air-dried. Bound dye was solubilized with 100 μl 100% DMSO for 20 minutes and the absorbance of the supernatants was measured at 544 nm using the FluoStar Optima (BMG LabTech) plate reader.

### Statistical analysis

Statistical analysis was performed using the Prism 5.0 software (GraphPad). In all cases the mean ± standard error of the mean (SEM) is shown unless otherwise stated. P values for statistical analysis were calculated using an one-tailed Mann-Whitney U test. Differences were considered statistically significant when p values were less than 0.05 (p<0.05) with a 95% confidence level.

### Ethics Statement

This study was carried out in strict accordance with the recommendations in the Australian Code of Practice for the Care and Use of Animals for Scientific Purposes. The PMoH protocol was approved by the Animal Ethics Committee of La Trobe University (permit Number: 09–14 B). The use of human liver tissue was approved by the Austin Health Human Research Ethics Committee (HREC approval number H2010/03979). Written informed consent was obtained from the patients for the use of their liver samples in research.

## Results

### Hepatocyte apoptosis is upregulated in the liver tissue of patients post-liver transplantation

To determine the effect of immunosuppressive agents on hepatocyte apoptosis in patients after liver transplantation, we analyzed the liver biopsies taken 6 to 26 months after liver transplantation of 10 patients who were transplanted for end-stage cirrhosis due to alcohol. Biopsies were performed in the setting of abnormal liver function tests post-liver transplantation. These specimens were compared to the liver biopsies taken from 9 subjects who were undergoing liver resection for colorectal metastasis (control group). The control patients did not have any underlying liver disease and the biopsies were taken from normal liver away from the site of metastatic tumour. Clinical and demographic data on these patients can be found in Table 1. Sections were examined for cleaved cytokeratin 18 (M30 CytoDEATH) and cleaved PARP, both markers of apoptosis.

**Table 1:** Patient demographics and clinical data for the 10 post-liver transplant patients and 9 control patients whose liver sections were analyzed for markers of apoptosis.

	Group	Gender	Age at Bx	Alb	Bili	ALT	INR	Biopsy A-Score	Biopsy F-Score	Evidence of Rejection	Months Post-Tx	Immunosupressants Used
1	Post-Tx	Male	46	41	14	94	1.0	1	0	Mild	15	CyA 150mg bd+ Aza 50mg daily
2	Post-Tx	Male	48	45	13	138	1.1	1	0	Mild	19	CyA 100mg bd
3	Post-Tx	Female	58	34	9	37	1.1	0	0	No	21	CyA 100mg bd+ Aza 50mg daily
4	Post-Tx	Male	58	31	25	194	1.0	2	0	Moderate	24	CyA 100mg bd+ MMF 500mg bd
5	Post-Tx	Male	58	31	10	166	0.9	1	0	Mild	26	Tac 4mg bd+ Pred 5mg daily
6	Post-Tx	Female	58	23	38	90	1.0	0	0	No	6	Tac 5mg bd+ MMF 1g bd
7	Post-Tx	Female	57	26	64	353	0.9	1	0	Mild	7	Tac 7mg bd+ Pred 10mg daily
8	Post-Tx	Male	51	26	46	101	0.9	0	0	No	6	Tac 4mg bd+ MMF 1g bd
9	Post-Tx	Male	50	33	19	147	1.1	1	0	No	12	Tac 3mg bd+ Myfortic 360mg bd
10	Post-Tx	Male	46	24	8	375	1.4	1	0	Mild	13	CyA 100mg bd+ Pred 10mg daily
1	Control	Male	41	28	79	87	1.3	0	0			
2	Control	Male	80	29	22	237	1.1	0	0			
3	Control	Female	30	30	10	114	1.1	0	0			
4	Control	Male	53	29	25	299	1.2	0	0			
5	Control	Female	56	22	78	318	1.2	0	0			
6	Control	Male	58	39	13	27	1.1	0	0			
7	Control	Male	73	43	14	45	1.1	0	0			
8	Control	Male	46	37	12	179	1.1	0	0			
9	Control	Female	43	45	12	88	1.2	0	0			

Post-Tx: Post-liver transplant; Bx: Liver biopsy; Alb: Serum albumin; Bili: Serum bilirubin; ALT: Serum alanine transaminase; INR: International Normalized Ratio; CyA: Cyclosporine; Aza: Azathioprine; Tac: Tacrolimus; Pred: Prednisolone; MMF: Mycophenolate mofetil.

**Fig 1:**
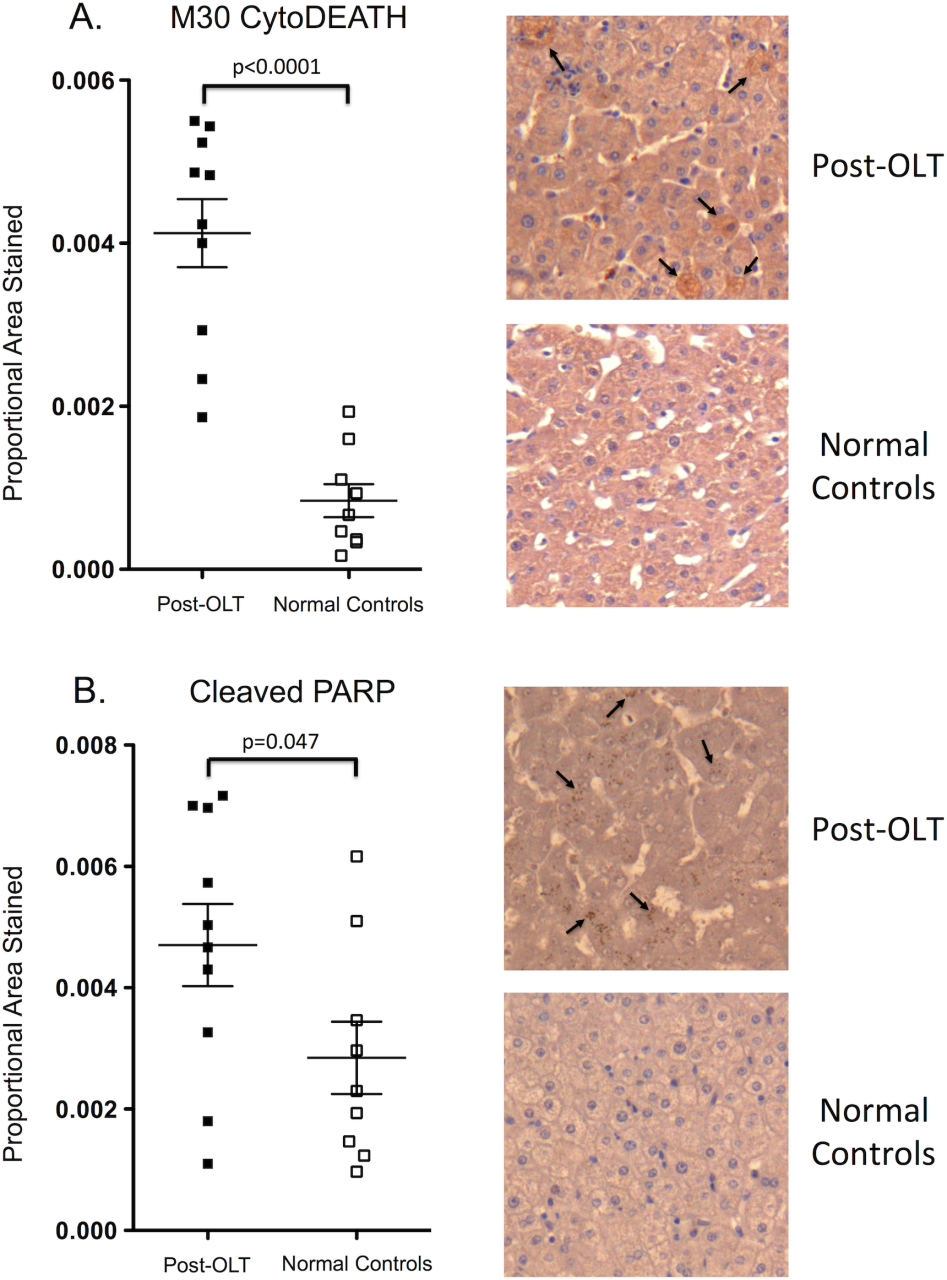
Increased hepatocyte apoptosis is seen after liver transplantation on immunohistochemistry staining of liver tissue. Photomicrographs show immunohistochemical staining of human liver sections for M30 CytoDEATH and cleaved PARP at 200x magnification, indicating increased levels of these markers of apoptosis in post-liver transplant patients compared to healthy subjects (arrows indicate positive staining cells). Graphs show the relative staining of these apoptotic markers in the liver of each patient assessed by computerized image capture quantification with (A) M30 CytoDEATH and (B) cleaved PARP results expressed as mean proportional area stained within the given area and error bars represent SEM.

The livers of post-liver transplant patients showed a 4.9-fold increase in M30 CytoDEATH staining compared to normal livers (p<0.0001) (Fig 1A). Liver biopsies from patients post-liver transplantation also demonstrated increased staining for cleaved PARP, with levels 1.7-fold higher than normal livers (p = 0.047) (Fig 1B). These results indicate that patients had elevated levels of hepatocyte apoptosis after liver transplantation. Therefore, to understand whether the immunosuppressive agents may have contributed to this increased apoptosis, we investigated the effects of immunosuppressant treatment on apoptosis in primary mouse hepatocytes in culture.

### Cyclosporine at therapeutic concentrations does not reduce cell viability or increase hepatocyte apoptosis

**Fig 2:**
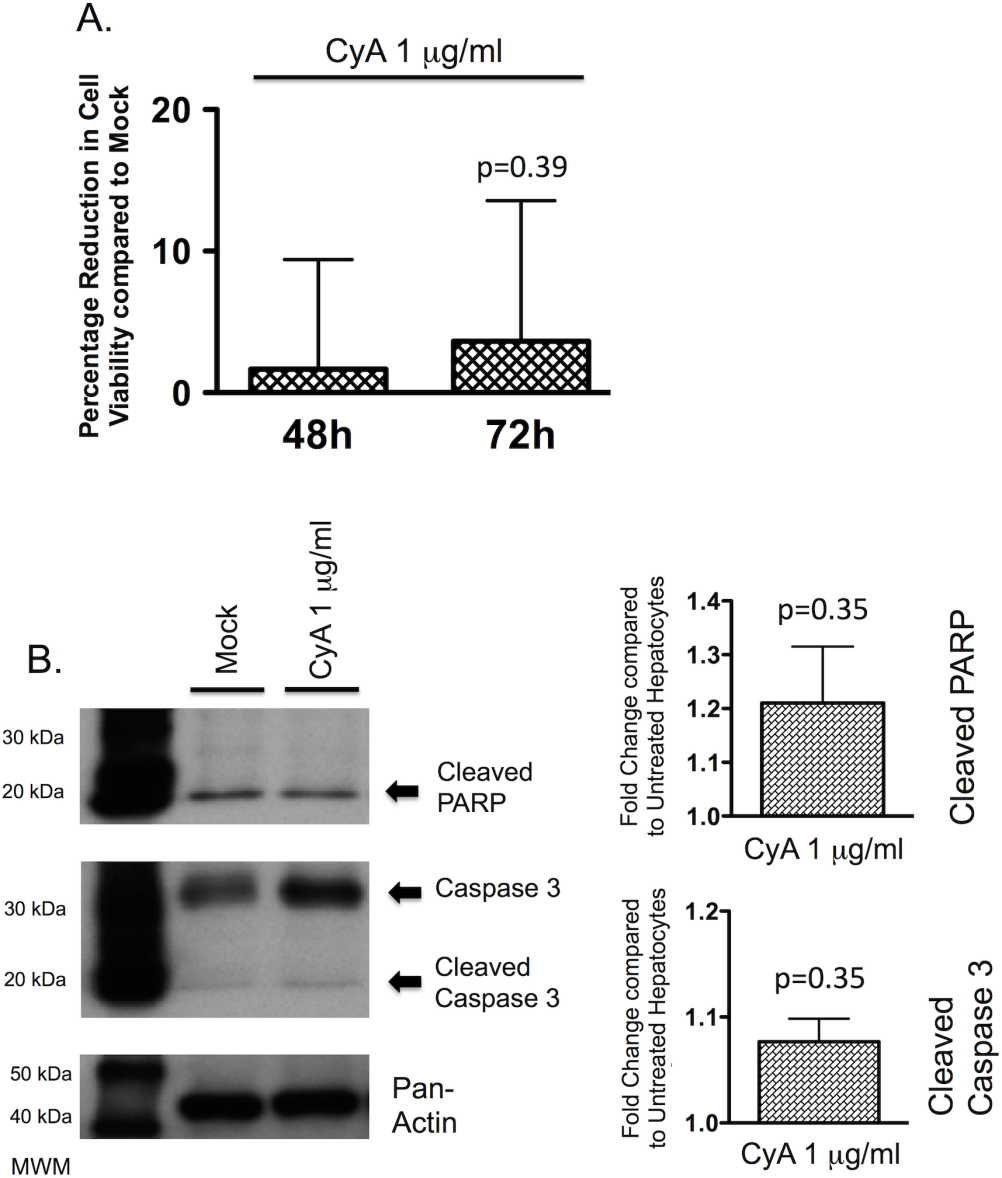
Cyclosporine at therapeutic concentrations does not increase hepatocyte cell death. (A) Percentage reduction in cell viability from crystal violet assays of PMoH treated with 1 μg/ml of cyclosporine compared to untreated cells. (B) Western blots of cleaved PARP and cleaved caspase 3 levels in PMoH treated with 1 μg/ml of cyclosporine compared to untreated cells. Graphs show fold change in cleaved PARP and cleaved caspase 3 levels relative to untreated cells. Each bar represents the average of 3 experiments and error bar represents SEM. P-values are compared to untreated hepatocytes.

The therapeutic range for cyclosporine post-liver transplantation is 0.7 to 1.3 μg/ml[13]. At a concentration of 1 μg/ml, cyclosporine had little effect on PMoH, only reducing cell viability by 3.6% (±SEM 9.9%) after 72 hours of treatment compared to untreated cells (p = 0.39) (Fig 2A).

We next investigated the effect of cyclosporine on hepatocyte apoptosis. In PMoH treated with 1 μg/ml of cyclosporine for 48 hours, neither cleaved PARP nor cleaved caspase 3 were significantly elevated compared to untreated cells, with cleaved PARP increased 1.2-fold (±SEM 0.11, p = 0.35), and cleaved caspase 3 increased 1.1-fold (±SEM 0.02, p = 0.35).

These results show that at the therapeutically relevant concentration used after liver transplantation, cyclosporine had no effect on cell viability or apoptosis in primary hepatocytes.

### Mycophenolate mofetil improves cell viability and decreases apoptosis in hepatocytes

**Fig 3:**
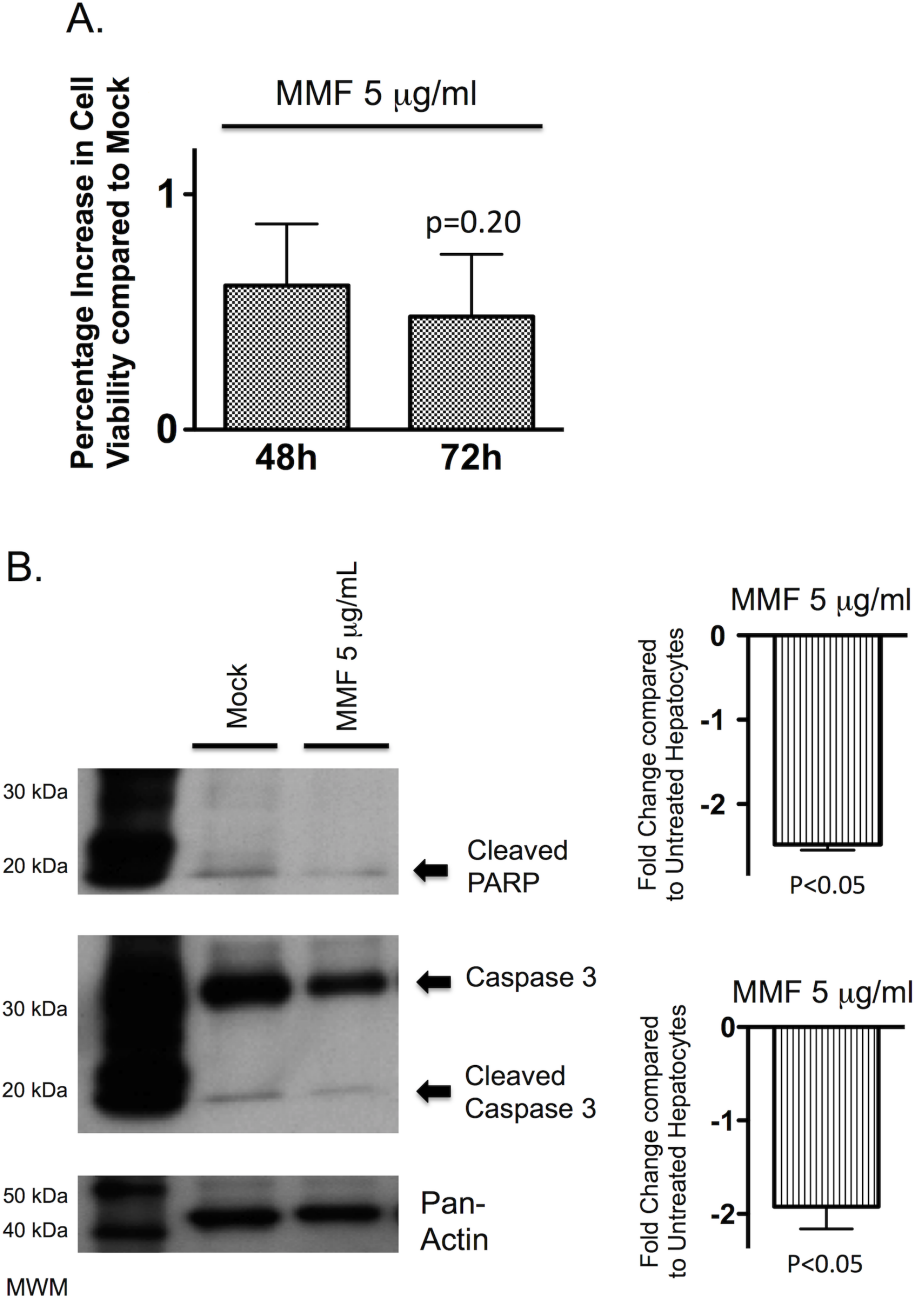
Mycophenolate mofetil reduces hepatocyte cell death in a dose-dependent manner. (A) Percentage increase in cell viability from crystal violet assays of PMoH treated with 5 μg/ml of MMF, compared to untreated cells. (B) Western blots of cleaved PARP and cleaved caspase 3 levels in PMoH treated with 5 μg/ml of MMF at 48 hours compared to untreated cells. Graphs show fold change in cleaved PARP and cleaved caspase 3 levels relative to untreated cells. Each bar represents the average of 3 experiments and error bar represents SEM. P-values are compared to untreated hepatocytes.

In PMoH treated with 5 μg/ml of MMF (a therapeutically relevant concentration), MMF was found to produce a marginal improvement in cell viability at 72 hours of treatment compared to untreated cells, but this did not achieve statistical significance (p = 0.20) (Fig 3A).

We then determined the effect of MMF on apoptosis in PMoH. Treatment of PMoH with 5 μg/ml MMF for 48 hours significantly decreased the level of cleaved PARP by 2.5-fold (±SEM 0.07, p<0.05) and reduced cleaved caspase 3 by 1.9-fold (±SEM 0.24, p<0.05) compared to untreated cells (Fig 3B). These results show that at the therapeutically relevant mean concentrations achieved post-liver transplantation, MMF improved cell viability and reduced apoptosis in primary hepatocytes.

### The combination of cyclosporine and mycophenolate mofetil reduces cell viability and increases hepatocyte apoptosis

Combinations of immunosuppressive drugs are more commonly used than individual drugs following liver transplantation. Therefore, to examine a more typical treatment scenario we assessed the effect of the combination of cyclosporine plus MMF on hepatocyte viability and apoptosis.

**Fig 4:**
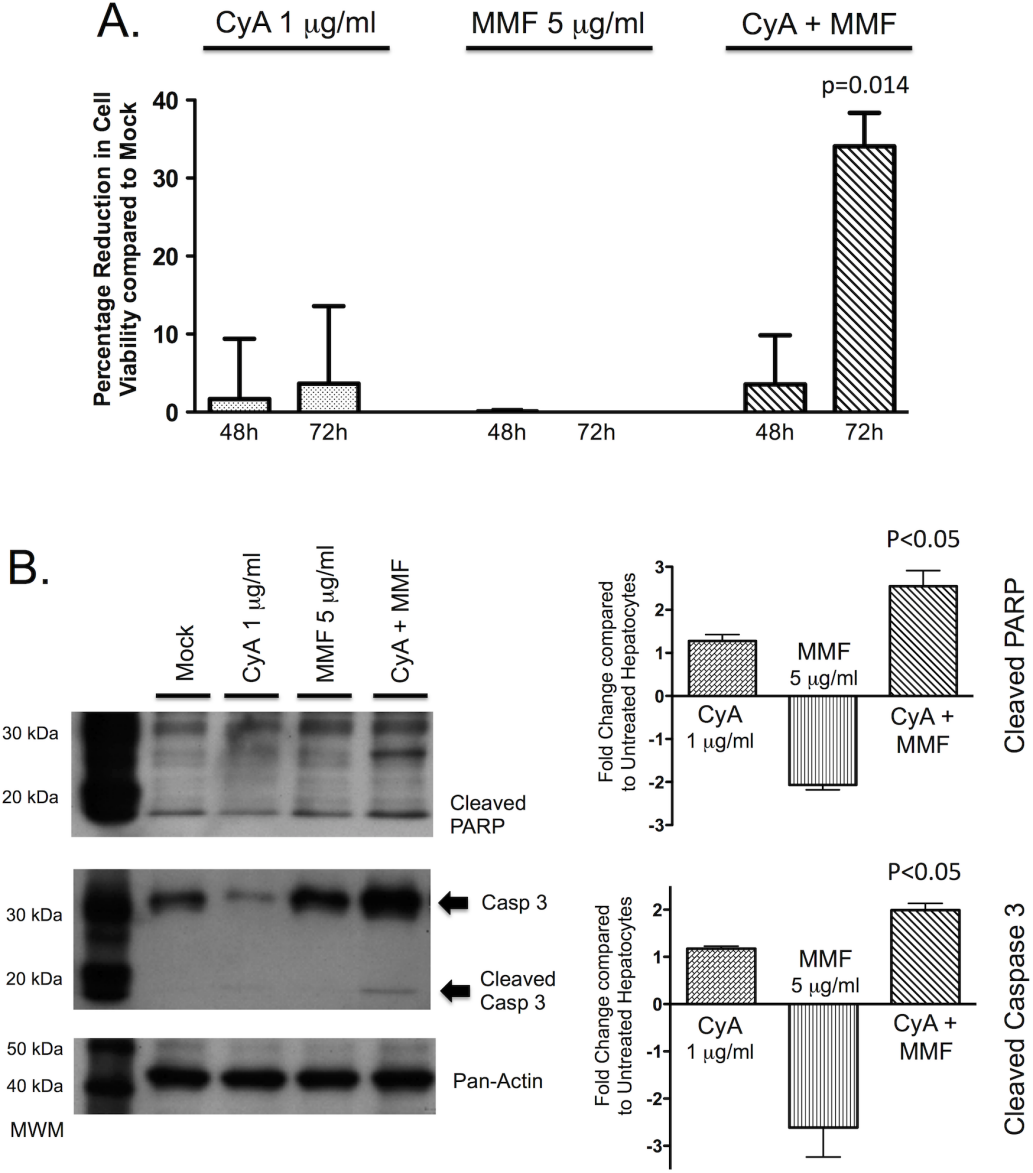
The combination of cyclosporine and mycophenolate mofetil enhances cell death in hepatocytes. (A) Percentage reduction in cell viability from crystal violet assays of PMoH treated with 1 μg/ml of cyclosporine and/or 5 μg/ml of MMF compared to untreated cells. (B) Western blots of cleaved PARP and cleaved caspase 3 levels in PMoH treated with 1 μg/ml of cyclosporine and/or 5 μg/ml of MMF compared to untreated cells. Graphs show fold change in cleaved PARP and cleaved caspase 3 levels in cyclosporine- and/or MMF-treated PMoH at 48 hours relative to untreated cells. Each bar represents the average of 3 experiments and error bar represents SEM. P-values are compared to untreated hepatocytes.

The combination of cyclosporine (1 μg/ml) and MMF (5 μg/ml) reduced PMoH cell viability by 34% (±SEM 4.3) at 72 hours compared to untreated cells (p = 0.014), an effect greater than cyclosporine alone (p = 0.029) (Fig 4A).

We then evaluated the effect of the combination of cyclosporine and MMF on hepatocyte apoptosis. Cleaved PARP was increased by 2.6-fold (±SEM 0.36, p<0.05), and cleaved caspase 3 was increased by 2.0-fold (±SEM 0.15, p<0.05) at 48 hours compared to untreated cells. This effect was also significantly greater than with cyclosporine treatment alone (p = 0.03 for cleaved PARP and p = 0.006 for cleaved caspase 3) (Fig 4B). These results show that the combination of cyclosporine and MMF reduced cell viability and increased apoptosis in primary hepatocytes.

### Tacrolimus at therapeutic concentrations does not reduce cell viability or increase apoptosis in hepatocytes

**Fig 5:**
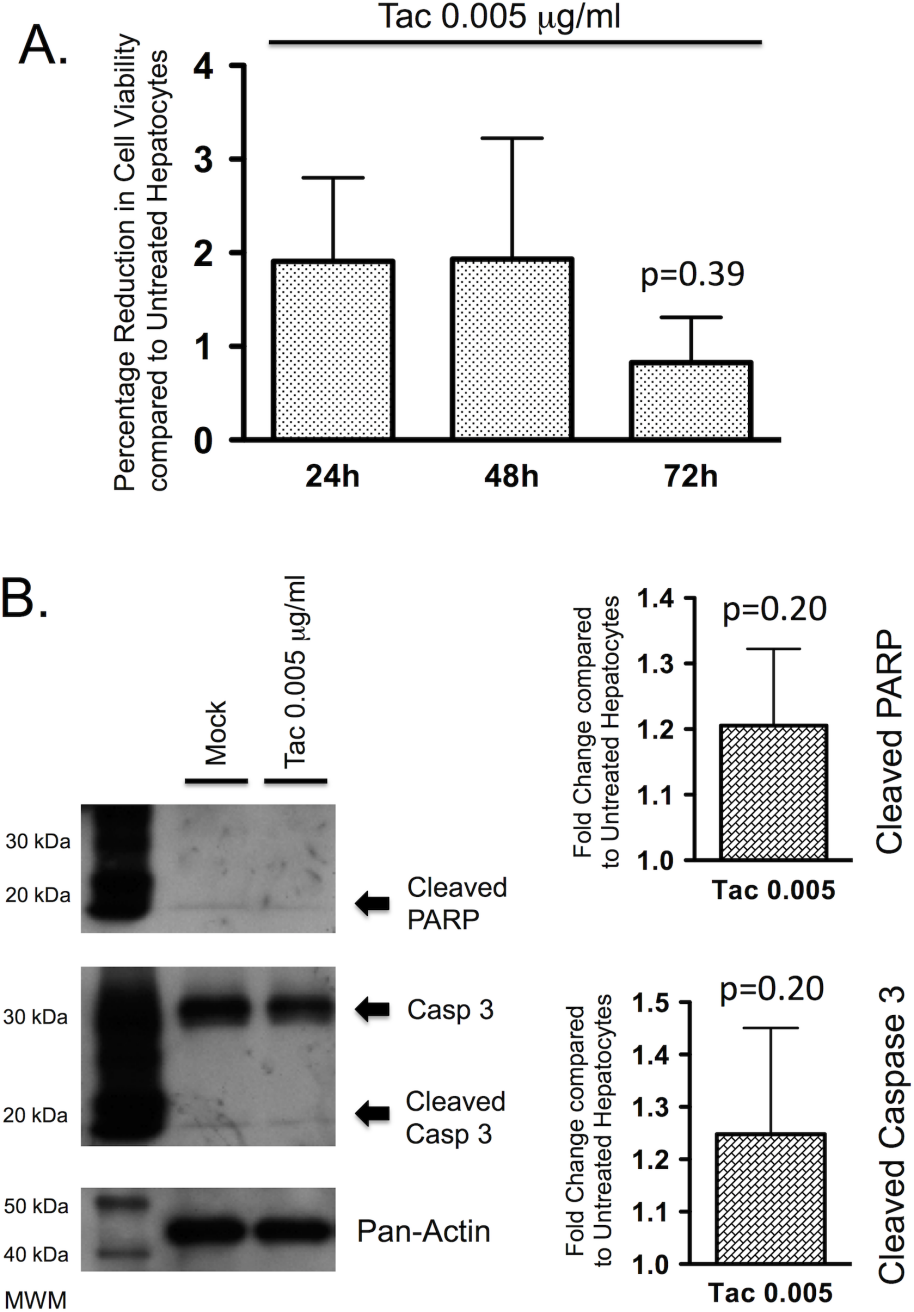
Tacrolimus at therapeutic concentrations does not affect hepatocyte cell death. (A) Percentage reduction in cell viability from crystal violet assays of PMoH treated with 0.005 μg/ml of tacrolimus at 24–72 hours compared to untreated cells. (B) Western blots of cleaved PARP and cleaved caspase 3 levels in PMoH treated with 0.005 μg/ml of tacrolimus at 48 hours compared to untreated cells. Graphs show fold change in cleaved PARP and cleaved caspase 3 levels in tacrolimus-treated PMoH relative to untreated cells. Each bar represents the average of 3 experiments and error bar represents SEM. P-values are compared to untreated hepatocytes.

For experiments with tacrolimus, a therapeutically relevant concentration of 0.005 μg/ml was used. At this concentration tacrolimus had no effect on PMoH cell viability with up to 72 hours of treatment compared to untreated cells (p = 0.39) (Fig 5A).

To determine the effect of tacrolimus on hepatocyte apoptosis, PMoH were treated with a therapeutic concentration of tacrolimus. At 0.005 μg/ml, the levels of cleaved PARP were not significantly altered compared to untreated cells at 48 hours (p = 0.20) (Fig 5B). A similar pattern was seen for cleaved caspase 3 where the levels in PMoH were not significantly increased by tacrolimus treatment at 48 hours compared to untreated cells (p = 0.20). These results show that tacrolimus does not affect cell viability or apoptosis in primary hepatocytes at the therapeutic concentrations achieved after liver transplantation.

### The combination of tacrolimus and mycophenolate mofetil reduces cell viability and increases hepatocyte apoptosis

**Fig 6:**
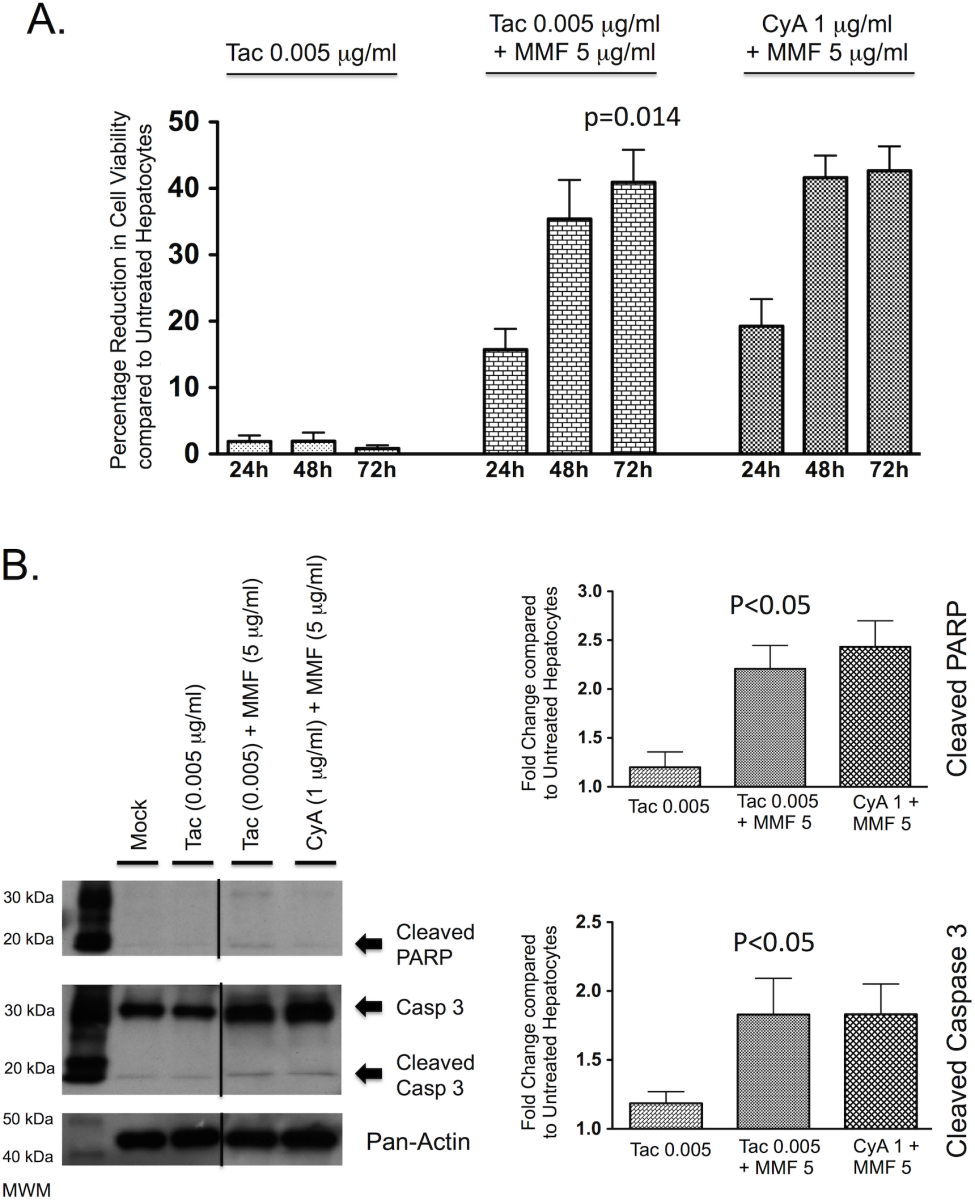
The combination of tacrolimus and MMF promotes cell death in hepatocytes. (A) Percentage reduction in cell viability from crystal violet assays of PMoH treated with 0.005 μg/ml of tacrolimus ± 5 μg/ml of MMF or 1 μg/ml of cyclosporine + 5 μg/ml of MMF at 24–72 hours compared to untreated cells. (B) Western blots of cleaved PARP and cleaved caspase 3 levels in PMoH treated with 0.005 μg/ml of tacrolimus ± 5 μg/ml of MMF or 1 μg/ml of cyclosporine + 5 μg/ml of MMF at 48 hours compared to untreated cells. Graphs show fold change in cleaved PARP and cleaved caspase 3 levels in tacrolimus/MMF-treated PMoH and cyclosporine/MMF-treated cells compared to untreated hepatocytes. Each bar represents the average of 3 experiments and error bar represents SEM. P-values are compared to untreated hepatocytes.

While at therapeutically relevant concentrations, tacrolimus alone was shown to have no effect on hepatocyte viability, treatment of PMoH with 0.005 μg/ml of tacrolimus combined with 5 μg/ml of MMF significantly decreased cell viability. At 72 hours of treatment with tacrolimus and MMF, cell viability was reduced by 41% compared to untreated PMoH (p = 0.014) (Fig 6A).

Similar to the cyclosporine/MMF combination, the combination of tacrolimus and MMF was found to promote hepatocyte apoptosis, with the levels of cleaved PARP and cleaved caspase 3 increased by 2.2-fold (±SEM 0.24, p<0.05) and 1.8-fold (±SEM 0.26, p<0.05) respectively in PMoH after 48 hours of treatment compared to untreated cells (Fig 6B). These findings indicate that treatment with the combination of tacrolimus and MMF reduces cell viability and increases hepatocyte apoptosis to a level similar to the combination of cyclosporine and MMF.

### Sirolimus at therapeutic concentrations does not affect hepatocyte viability or apoptosis

**Fig 7:**
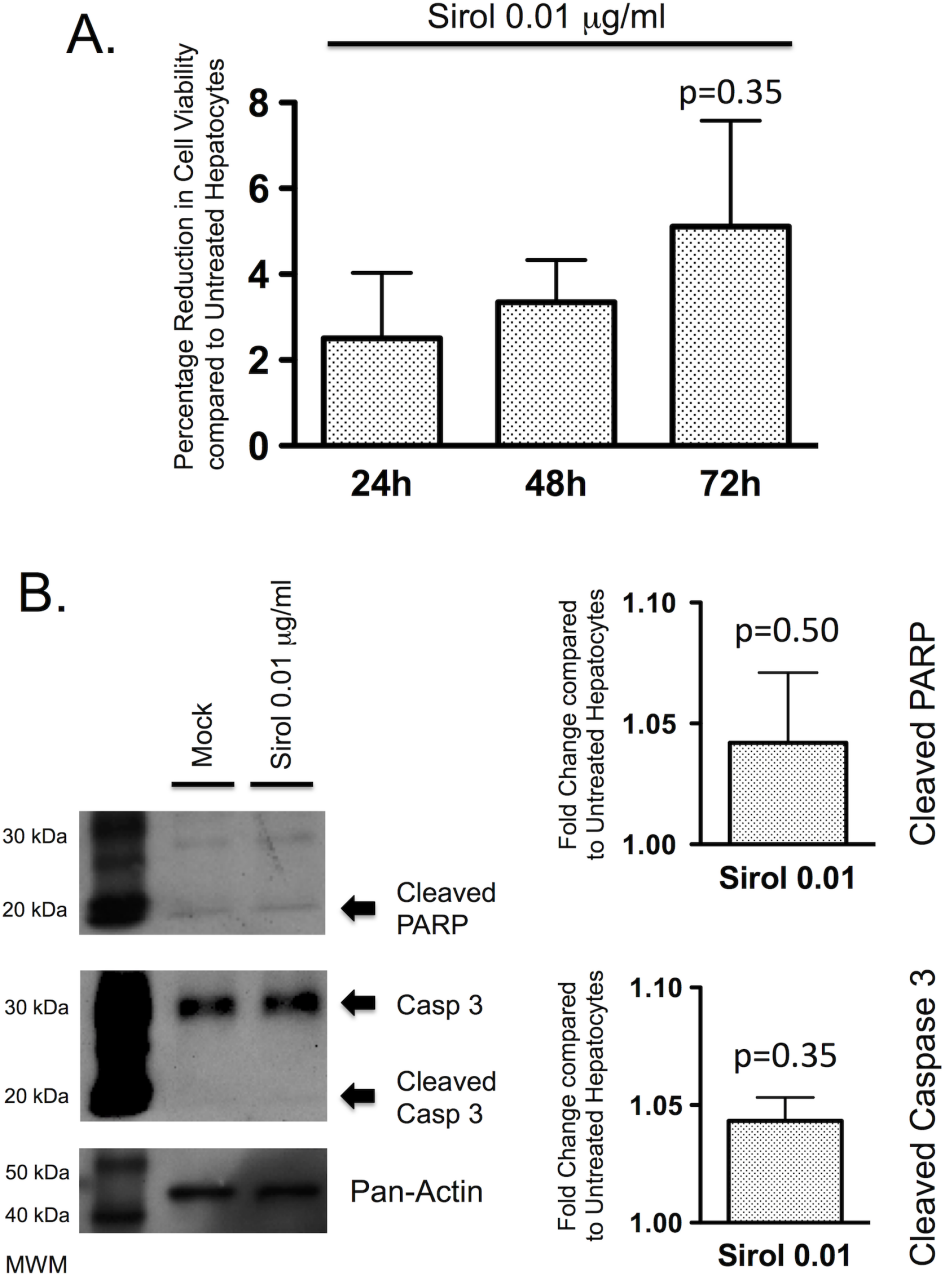
Sirolimus at therapeutic concentrations does not increase hepatocyte cell death. (A) Percentage reduction in cell viability from crystal violet assays of PMoH treated with 0.01 μg/ml of sirolimus at 24–72 hours compared to untreated cells. (B) Western blots of cleaved PARP and cleaved caspase 3 levels in PMoH treated with 0.01 μg/ml of sirolimus at 48 hours compared to untreated cells. Graphs show fold change in cleaved PARP and cleaved caspase 3 levels in sirolimus-treated PMoH compared to untreated cells. Each bar represents the average of 3 experiments and error bar represents SEM. P-values are compared to untreated hepatocytes.

Sirolimus, at a therapeutically relevant concentration of 0.01 μg/ml had no effect on cell viability in PMoH after 72 hours of treatment compared to untreated cells (p = 0.35) (Fig 7A).

To evaluate the effect of sirolimus on hepatocyte apoptosis, we treated PMoH with a therapeutic concentration of sirolimus. In PMoH treated with 0.01 μg/ml of sirolimus, both cleaved PARP and cleaved caspase 3 levels were not significantly different from untreated cells at 48 hours (p = 0.50 for cleaved PARP and p = 0.35 for cleaved caspase 3) (Fig 7B). These results demonstrate that, as with tacrolimus, a therapeutic concentration of sirolimus has no effect on cell viability or apoptosis of primary hepatocytes.

### The combination of sirolimus and mycophenolate mofetil had minimal effect on cell viability or apoptosis in hepatocytes

As sirolimus is a newer immunosuppressive agent that is now being used to substitute cyclosporine or tacrolimus in the setting of calcineurin toxicity, the combination of sirolimus and MMF may become more commonplace in the future and the effect of this combination on hepatocyte cell death warrants investigation.

**Fig 8:**
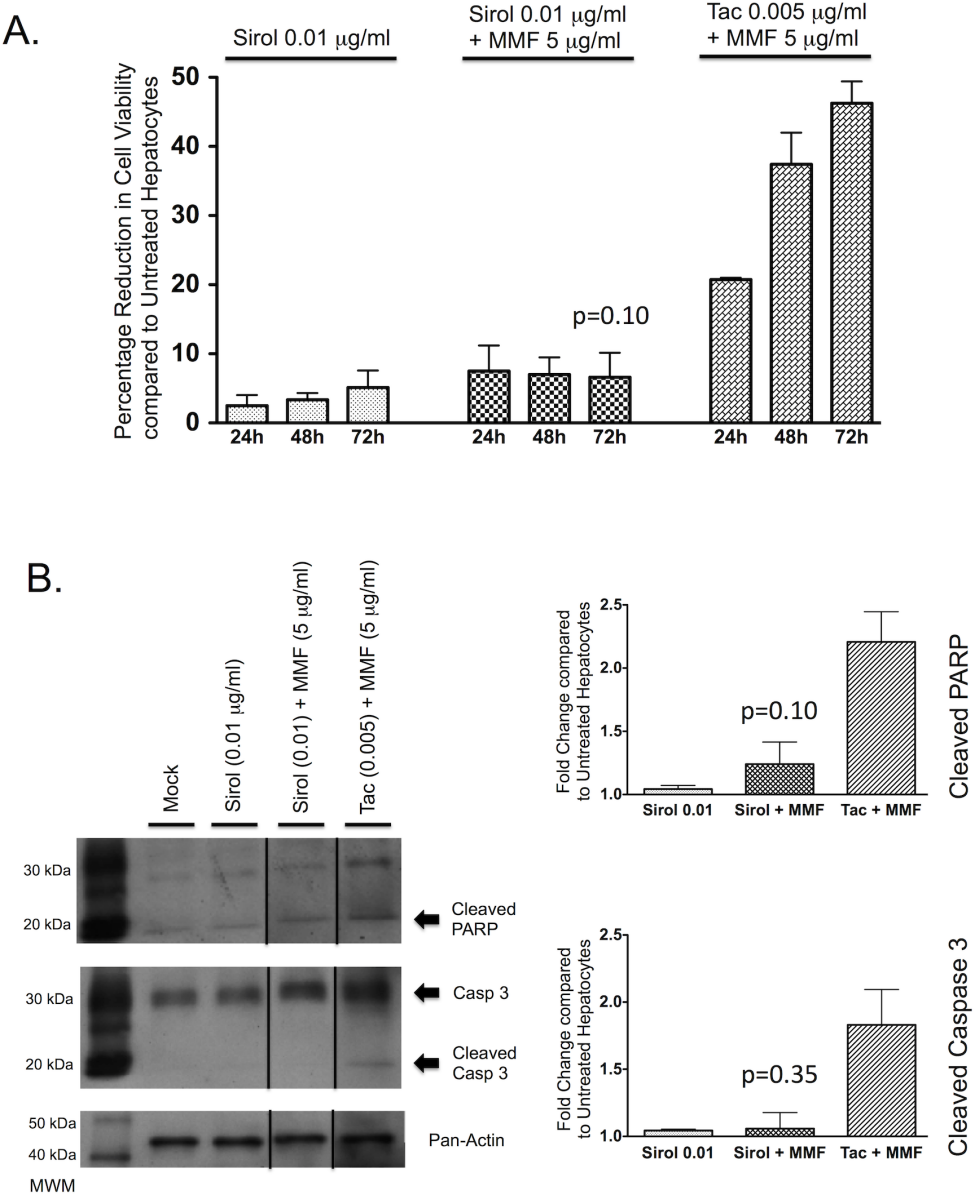
The combination of sirolimus and mycophenolate mofetil has no significant effect on cell death in hepatocytes. (A) Percentage reduction in cell viability from crystal violet assays of PMoH treated with 0.01 μg/ml of sirolimus ± 5 μg/ml of MMF or 0.005 μg/ml of tacrolimus + 5 μg/ml of MMF at 24–72 hours compared to untreated cells. (B) Western blots of cleaved PARP and cleaved caspase 3 levels in PMoH treated with 0.01 μg/ml of sirolimus ± 5 μg/ml of MMF or 0.005 μg/ml of tacrolimus + 5 μg/ml of MMF at 48 hours compared to untreated cells. Graphs show fold change in cleaved PARP and cleaved caspase 3 levels in sirolimus/MMF-treated PMoH and tacrolimus/MMF-treated cells compared to untreated hepatocytes. Each bar represents the average of 3 experiments and error bar represents SEM. P-values are compared to untreated hepatocytes.

The combination of sirolimus and MMF did not significantly decrease cell viability in PMoH over 72 hours compared to untreated cells (p = 0.10) (Fig 8A). In addition, this combination did not significantly increase either cleaved PARP (p = 0.10) or cleaved caspase 3 (p = 0.35) in primary hepatocytes compared to untreated cells after 48 hours of treatment (Fig 8B). These results show that in contrast to the combinations of cyclosporine/MMF and tacrolimus/MMF, sirolimus plus MMF does not reduce cell viability or increase apoptosis in primary hepatocytes.

## Discussion

Programmed cell death may occur by one of a number of well described pathways including autophagy, which is a catabolic process mediated by autophagosomes; necroptosis, a programmed form of inflammatory cell death that is caspase-independent; and apoptosis, where two separate pathways (extrinsic and intrinsic) converge upon an executioner caspase cascade producing blebbing, cell shrinkage, nuclear fragmentation, chromatin condensation and ultimately cell destruction. There is increasing data implicating hepatocyte apoptosis as a driving force for fibrogenesis in a range of different causes of liver injury[14]. Apoptotic hepatocytes are engulfed and cleared by both Kupffer cells and hepatic stellate cells (HSCs). The uptake of apoptotic bodies by HSCs results in their activation and secretion of TGF-β[15], a key factor in stimulating liver fibrosis[16]. Kupffer cells that have ingested apoptotic hepatocytes also secrete TGF-β[17], further promoting the pro-fibrogenic response by activated HSCs.

We have shown that in the livers of patients who have undergone liver transplantation, the levels of hepatic apoptotic markers are increased compared to the livers of normal subjects. Although cellular rejection may in part account for the increased hepatocyte apoptosis seen, only 6 of the 10 post-transplant patients had evidence of rejection, and for those in whom it was present the rejection was mild. Furthermore, the increased apoptosis was seen consistently in all post-transplant patients regardless of whether there was cellular rejection. As such, the immunosuppressive agents used following transplantation may themselves have cytotoxic and pro-apoptotic effects that could promote hepatocyte apoptosis and thereby contribute to inflammation and the pathogenesis of liver graft injury following transplantation. However there have been few studies of the effect of commonly used immunosuppressive agents on hepatocyte viability. We therefore investigated the impact of these drugs on hepatocyte viability and apoptosis, and found that in primary hepatocytes, these immunosuppressive drugs in combination reduced cell viability and enhance hepatocyte apoptosis. Thus the data from the primary hepatocyte experiments support our claim that the pro-apoptotic effect observed in the human livers was the result of the immunosuppressive agents.

The calcineurin inhibitors tacrolimus and cyclosporine remain the most commonly used immunosuppressive agents in liver transplantation. Studies of the effects of these immunosuppressants on cell death in non-liver cell types have yielded conflicting results. Cyclosporine has been found to cause apoptosis of renal vascular endothelial cells[7], and fibrosis of the renal tubulointerstitium by upregulating TGF-β expression[18], raising concerns that similar effects may occur in the liver. Indeed, cyclosporine has been noted to promote hepatocyte expression of Bak, a pro-apoptotic protein, in a rat model of liver injury[19]. Conversely, cyclosporine has also been shown to prevent apoptosis of human gingival fibroblasts by inhibiting Bax and upregulating anti-apoptotic Bcl-2[20], and inhibiting cytochrome c release in human platelets and rat vascular endothelial cells[21]. Like cyclosporine, tacrolimus has been noted to have both pro-apoptotic and anti-apoptotic effects in various non-hepatic cell lines. Tacrolimus has been shown to promote the generation of reactive oxygen species, mitochondrial dysfunction and apoptosis in Jurkat T-cells[8]. By contrast, tacrolimus has an anti-apoptotic effect in human islet cells treated with pro-inflammatory cytokines, causing a reduction in TNF-α and down-regulation of caspase-3[22]. In the current study we found that at therapeutically relevant concentrations, neither cyclosporine nor tacrolimus promoted hepatocyte cell death.

In the early post transplant period MMF is most commonly used in combination with cyclosporine or tacrolimus. However patients with calcineurin antagonist induced renal dysfunction are commonly switched to MMF as the primary immunosuppressive drug. MMF treatment has been found to increase apoptosis of epithelial cells in the upper gastrointestinal tract and colon[10], as well as promote apoptosis in human pancreatic islet cells[23], but MMF has also been noted to reduce apoptosis of renal tubular epithelial, glomerular and interstitial cells[24]. In contrast to the other drugs studied, MMF treatment appeared to improve cell viability and reduce apoptosis in primary hepatocytes suggesting that apart from its renal sparing effects[25], the switch to MMF-based therapy may also have a hepatoprotective effect compared to other immunosuppressive drugs.

The combination of tacrolimus or cyclosporine and MMF is increasingly being used as the cornerstone of post-liver transplantation immunosuppresssion[26], with tacrolimus being more commonly used since this drug has been shown to be superior to cyclosporine in a number of clinical studies[2]. Importantly, the hepatoprotective effect of MMF was lost when the drug was combined with either cyclosporine or tacrolimus. In this setting MMF was found to enhance cell death to a greater level than we observed with either cyclosporine or tacrolimus alone. We found that the combination of cyclosporine and MMF enhanced hepatocyte apoptosis as much as tacrolimus plus MMF at therapeutically relevant concentrations. How these calcineurin inhibitors interact with MMF to exert this toxic effect within hepatocytes is currently unknown. Why combination therapy results in hepatocyte injury when cyclosporine or tacrolimus used alone does not is currently unknown, but perhaps the treatment with a single immunosuppressive agent is insufficient to cause toxicity. We know that when tested in concentrations well above the therapeutic range (10 to 100 fold) the individual drugs do induce hepatocyte apoptosis (results not shown). It is therefore possible that when these agents are used in combination at therapeutic concentrations, there may be an additive toxic effect that is sufficient to result in hepatocyte injury.

Although the combination of a calcineurin inhibitor and MMF is currently a commonly employed regimen following liver transplantation, sirolimus is used to substitute for either cyclosporine or tacrolimus in the setting of calcineurin antagonist-induced adverse effects such as renal impairment and diabetes mellitus[27], and the use of sirolimus and MMF combination therapy may become commonplace in the future. Sirolimus has been found to induce apoptosis in acute lymphoblastic leukemia cells[28] and vascular smooth muscle cells[9]. However, sirolimus has also been shown to reduce liver fibrogenesis, improve liver function and enhance survival in rats with experimental liver fibrosis[29]. In our study, we have found that sirolimus at the post-liver transplant therapeutic concentration did not promote hepatocyte cell death. Furthermore, sirolimus appears to be less toxic than either cyclosporine or tacrolimus when combined with MMF, with our results showing that at the therapeutic concentrations, treatment with the sirolimus/MMF combination resulted in a smaller reduction in cell viability and hepatocyte apoptosis than the tacrolimus/MMF combination.

Our results indicate that the current commonly used maintenance immunosuppressive drug regimens employed after liver transplantation of cyclosporine/MMF and tacrolimus/MMF may enhance hepatocyte cell death, and thus may contribute to increased liver fibrosis that occurs in a proportion of liver transplant recipients. At therapeutically relevant concentrations, neither cyclosporine nor tacrolimus monotherapy promoted hepatocyte cell death. Whether the threshold concentration at which these effects are observed may be lower in the presence of other common post transplant injurious stimuli such as recurrent viral hepatitis or biliary disease will require further investigation. Future studies to further investigate the *in vitro* findings of immunosuppressant toxicity in hepatocytes could examine liver sections obtained from mice treated with immunosuppressant combinations. An important finding from our study is the lack of toxicity of sirolimus and of the combination of sirolimus and MMF. This suggests that this immunosuppressive regimen may be beneficial in reducing liver allograft injury in patients following liver transplantation and warrants further investigation.
